# Efficacy and Safety of Medicines Targeting Neurotrophic Factors in the Management of Low Back Pain: Protocol for a Systematic Review and Meta-analysis

**DOI:** 10.2196/22905

**Published:** 2021-01-22

**Authors:** Rodrigo R N Rizzo, Michael C Ferraro, Michael A Wewege, Aidan G Cashin, Hayley B Leake, Edel T O’Hagan, Matthew D Jones, Sylvia M Gustin, James H McAuley

**Affiliations:** 1 School of Medical Sciences University of New South Wales Sydney Australia; 2 Centre for Pain IMPACT Neuroscience Research Australia Sydney Australia; 3 Prince of Wales Clinical School University of New South Wales Sydney Australia; 4 IIMPACT in Health University of South Australia Adelaide Australia; 5 School of Psychology University of New South Wales Sydney Australia

**Keywords:** back pain, analgesics, drug therapy, monoclonal antibodies, nerve growth factor, review, meta-analysis, antibodies, pain, back, growth factor, disability, sciatica

## Abstract

**Background:**

Low back pain (LBP) is the leading cause of years lived with disability worldwide. Most people with LBP receive the diagnosis of nonspecific LBP or sciatica. Medications are commonly prescribed but have limited analgesic effects and are associated with adverse events. A novel treatment approach is to target neurotrophins such as nerve growth factor (NGF) to reduce pain intensity. NGF inhibitors have been tested in some randomized controlled trials (RCTs) in recent years, showing promise for the treatment of chronic LBP; however, their efficacy and safety need to be evaluated to guide regulatory actions.

**Objective:**

The aim of this study is to evaluate the efficacy and safety of medicines targeting neurotrophins in patients with LBP and sciatica.

**Methods:**

In this systematic review, we will include published and unpublished records of parallel RCTs and the first phase of crossover RCTs that compare the effects of medicines targeting neurotrophins with any control group. We will search the CENTRAL, MEDLINE, Embase, CINAHL, ClinicalTrials.gov, EU Clinical Trials Register, and WHO International Clinical Registry Platform databases from inception. Pairs of authors will independently screen the records for eligibility, and we will independently extract data in duplicate. We will conduct a quantitative synthesis (meta-analysis) with the studies that report sufficient data and compare the medicines of interest versus placebo. We will use random-effects models and calculate estimates of effects and heterogeneity for each outcome. We will assess the risk of bias for each study using the Cochrane Collaboration tool, and form judgments of confidence in the evidence according to GRADE recommendations. We will use the PRISMA statement to report the findings. We plan to conduct subgroup analyses by condition, type of medication, and time point. We will also assess the impact of a potential new trial on an existing meta-analysis. Data from studies that meet inclusion criteria but cannot be included in the meta-analysis will be reported narratively.

**Results:**

The protocol was registered on the Open Science Framework on May 19, 2020. As of December 2020, we have identified 1932 records.

**Conclusions:**

This systematic review and meta-analysis will assess the evidence for the efficacy and safety of NGF inhibitors for pain in patients with nonspecific LBP and sciatica. The inclusion of new studies and unpublished data may improve the precision of the effect estimates and guide regulatory actions of the medications for LBP and sciatica.

**Trial Registration:**

Open Science Framework; https://osf.io/b8adn/

**International Registered Report Identifier (IRRID):**

DERR1-10.2196/22905

## Introduction

Low back pain (LBP) is the most prevalent musculoskeletal condition globally and the leading cause of years lived with disability [1,2]. Approximately 5%-10% of people with LBP have radiating pain that follows a dermatomal pattern, which leads to the diagnosis of sciatica [3]. For 90% of people with LBP, the nociceptive source cannot be reliably identified, and the condition is classified as nonspecific LBP [4,5]. A substantial number of people with nonspecific LBP and sciatica (28%) experience severe levels of disability [2], and 65% of individuals still have some level of continuing or fluctuating pain 3 months after the first episode [6,7].

Analgesic medicines are commonly prescribed for LBP [8,9]. There is widespread use of acetaminophen, nonsteroidal anti-inflammatory drugs (NSAIDs), corticosteroids, skeletal muscle relaxants, opioid analgesics, antidepressants, benzodiazepines, and antiepileptic drugs (eg, gabapentin) for all types of acute and chronic LBP [8]. These medicines have different proposed biological targets. Acetaminophen and NSAIDs likely exert their analgesic and anti-inflammatory actions by reducing prostaglandin synthesis [10]. Opioids mimic the actions of endogenous opioid peptides by interacting with presynaptic receptors; this interaction reduces neuronal excitability and inhibits nociceptive neurotransmitters in the central nervous system [11]. Antidepressants often involve reuptake inhibition of neurotransmitters involved in descending inhibition transmission of pain [12]. Gabapentin and pregabalin decrease the release of nociceptive transmitters by binding to calcium channels at different parts of the central nervous system [13]. In general, these medicines have limited analgesic effects and are often associated with negative side effects [14]. For example, opioids are associated with constipation, nausea, vomiting, itching, and somnolence, and only a minority of people have substantial pain relief [15-17]. Accordingly, research has focused on the investigation of other mechanisms and novel analgesic agents for chronic pain [18].

Neurotrophins constitute a group of structurally related proteins that include nerve growth factor (NGF), brain-derived neurotrophic factor (BDNF), glial cell-derived neurotrophic factor (GDNF), neurotrophin-3, and neurotrophin-4 [19,20]. These molecules are expressed at different levels in the peripheral and central nervous system after injury, inflammation, or exposure to a noxious stimulus, and contribute to the persistence of pain [19,21,22]. Preclinical studies have demonstrated that levels of NGF are elevated in a variety of chronic pain conditions (eg, interstitial cystitis, prostatitis, arthritis, pancreatitis, chronic headaches, cancer pain, diabetic neuropathy, sciatica, and other noncancer pain) [23-25]. Experimental studies have demonstrated that NGF and BDNF can maintain peripheral and central pain sensitization [22,26].

Approaches have been developed to target neurotrophins, including anti-NGF agents, which are in the most advanced phases of clinical testing. NGF inhibitors (or anti-NGF) are considered biological agents because they are harvested from a living system instead of being synthesized chemically as is the case for conventional analgesics [27]. Biologic drugs are relatively large molecules, which may have more precise mechanisms compared to conventional medicines and result in fewer adverse effects compared to opioids [27]. However, biologic agents interfere with key molecules in the human physiology, which may produce adverse effects [28]. Anti-NGF medicines have been tested in some chronic pain conditions, particularly LBP and osteoarthritis [23,25,29]. Anti-NGF medicines are commonly administered to people with chronic pain via multiple subcutaneous and intravenous injections, delivered at least 1 week apart [30,31]. Anti-NGF medicines may target novel mechanisms for pain relief and reduce some of the risks associated with opioids, such as medication addiction, misuse, dependence, and other side effects [18]. Anti-NGF medicines have not yet received approval from the US Food and Drug Administration (FDA), although the FDA recently accepted regulatory submission for tanezumab, an anti-NGF medicine, to treat patients with osteoarthritis [32]. The goal date for the FDA to make a decision is December 2020. The last systematic review with a meta-analysis that investigated the effect of anti-NGF medicines for LBP was published in 2014 [30]. Since then, several randomized controlled trials (RCTs) with large numbers of participants have been conducted. Therefore, this systematic review will evaluate the effect and safety of medicines targeting neurotrophins in patients with LBP.

## Methods

### Protocol Registration and Design

The protocol for this review is registered on the Open Science Framework (osf.io/b8adn). This protocol follows the PRISMA-P (Preferred Reporting Items for Systematic Reviews and Meta-Analysis Protocols) reporting guidelines [33,34].

### Eligibility Criteria

#### Study Design

We will include published and unpublished records of parallel RCTs and the first phase of crossover RCTs that compare the effect of medicines targeting neurotrophins with any control group. We will include secondary records of included trials (reporting additional results from the same trial) when authors provide additional results on outcomes or time points of interest. We will not include enriched-enrollment or cluster RCTs, or observational studies. We will include records reported in any language, except those for which a translator cannot be obtained.

#### Participants

We will include studies on adults aged 18 years or over diagnosed with LBP or sciatica of any duration. LBP is defined as a primary area of pain between the 12th rib and gluteal fold with or without leg pain [35]. Sciatica is defined as radiating pain in one leg combined with a positive result on one or more neurological tests indicating nerve root tension or neurological deficit [3]. We will consider the following pain duration: acute (from the first episode to 6 weeks), subacute (from 6 weeks to 12 weeks), and chronic (12 weeks or more) [36]. We will include studies that randomized participants with heterogeneous pain conditions (eg, hip osteoarthritis and LBP) only if we are able to obtain separate data for participants with LBP. Participants may be experienced or naïve to the trial intervention. We will exclude interventions that are combined with surgery; however, studies that investigated the effect of medicines targeting neurotrophins to prevent surgical/operative interventions will be included in the review. We will exclude LBP that is attributed to a specific pathology other than sciatica, such as infection, neoplasm, metastasis, inflammatory disease (eg, ankylosing spondylitis), or fractures.

#### Interventions

For this review, we will include any type of agent designed to target neurotrophins for the management of LBP. We expect to identify a larger number of medicines that target NGF than those targeting other neurotrophins. The medicines do not need to be listed on the World Health Organization Anatomical Therapeutic Chemical system or licensed for current use, because biologic drugs are new to the management of pain and still need to show their efficacy and safety. Medicines may be delivered as monotherapy or combination therapy via any route of administration (eg, subcutaneous, epidural, oral, intravenous). We will consider separate doses given that biologic agents are not FDA-approved and there may be a dose-response effect.

#### Comparators

We will include studies comparing the effect of a medicine targeting neurotrophins to any of the following interventions: (1) placebo/sham medicine, (2) waiting list or no treatment, (3) another dose or type of medicine targeting neurotrophins, and (4) other medicines. We will not exclude studies that assign nonpharmacological cointerventions to one or more of the intervention arms. We define the placebo intervention as any drug intervention that does not contain an active ingredient. We consider that waiting list or no treatment includes continuation of usual care or being placed on a waitlist.

### Outcome Measures

#### Primary Outcomes

The primary outcomes are pain intensity and safety. Pain intensity reported in the lower back can be measured by any self-report scale such as the visual analog scale (VAS), numeric rating scale (NRS), or a rating scale within a composite measure of pain (eg, McGill Pain Questionnaire). We will assume that ordinal scales exhibit continuous properties [37].

Safety is defined as the number of participants who experience an adverse effect during the treatment period [38].

#### Secondary Outcomes

The secondary outcomes include leg pain, back-specific function, and harm. Leg pain is defined as pain intensity in the leg due to sciatica as measured by any self-report scale. Leg pain has been used as a separate outcome for sciatica in previous studies [39].

Back-specific function can be measured by any self-report scale such as the Roland Morris Disability Questionnaire (RMDQ), Oswestry Disability Index (ODI), or a rating scale within a composite measure (eg, LBPRS-DI) [40].

Harm is defined as the number of participants who experience a serious adverse effect during the treatment period. A serious adverse event is defined according to the USA FDA as any event that results in death, is life threatening, requires inpatient hospitalization or causes prolongation of existing hospitalization, results in persistent or significant disability/incapacity, may have caused a congenital anomaly/birth defect, or requires intervention to prevent permanent impairment or damage [38].

#### Time Points of Outcomes Measures

We will consider outcomes measured at the time point closest to (1) 4 weeks, (2) 12 weeks, (3) 24 weeks, and (4) 48 weeks after randomization, regardless of the number of injections administered in the study.

### Search Strategy

We will search the following electronic databases from inception to present: MEDLINE (Ovid), Embase (Ovid), CINAHL (EBSCO), Cochrane Central Register of Controlled Trials (CENTRAL), ClinicalTrials.gov, EU Clinical Trials Register, and WHO International Clinical Trial Registry Platform. We will manually search the reference lists of included studies and previous reviews to identify additionally eligible studies.

We will combine terms related to RCTs, LBP, and spinal disorders, and interventions of interest as recommended by the Guideline for Systematic Reviews in the Cochrane Back and Neck Pain Group [41] and Cochrane Handbook [42]. We reviewed previous studies and conducted a preliminary search to inform the search terms for drugs that target neurotrophic factors. The search strategy for MEDLINE (Ovid) is detailed in Multimedia Appendix 1.

### Study Selection and Management

We will upload the records to Covidence [43], which will apply an automatic deduplication function to remove remaining duplicate records. Two reviewers will independently screen studies for title and abstract eligibility. We will retrieve full texts of all records where exclusion could not be determined from title and abstract screening. Two reviewers (RR and one or more reviewers) will independently screen the full text of these records for eligibility. Reviewers will give reasons for exclusion, and disagreements will be resolved through discussion or arbitration from a third author (JM) if required. We will search and identify related records (eg, other journal articles, conference abstracts, and trial registries). We will link related records for each included trial and follow this order for data extraction: (1) journal article of the trial, (2) conference abstract, (3) trial registration, (4) other records. We will summarize the search process using an adapted PRISMA flow diagram [44].

### Data Management and Extraction

Two reviewers (RR and one or more reviewers) will independently extract and enter data from the included trials into standardized Microsoft Excel spreadsheets. Disagreements between reviewers will be resolved through discussion or arbitration from a third author (JM) if necessary.

We will extract data for (1) trial characteristics, including country, setting, number of trial sites, sample size, and study duration; (2) participants*,* including diagnosis, duration of LBP, age, male/female ratio, arm-level pain intensity at baseline (mean and SD), and experience or naivety with the trial intervention; (3) interventions, including medicine tested, control, duration of intervention, dosage regimen, routes of administration, and usage of rescue medication; and (4) outcomes, including type and dimensions of the scale/measure and the time from randomization at which the outcome data were measured. For adverse events, we will extract the definition used in each study, and extract the type and number of adverse events in each intervention group.

If studies report more than one measure for pain, we will prioritize extraction in the following order: 100-mm VAS, 10-cm VAS, 11-point NRS, rating scale for pain intensity from a composite measure of pain (eg, McGill Pain Questionnaire), ordinal scale. If studies report more than one measure for function, we will prioritize extraction in the following order: ODI, RMDQ, rating scale for functional ability from a composite measure, ordinal scale. For both pain intensity and function, we will preferentially extract the outcome score and measure of variance at the end of treatment (or closest time point) for each group, followed by the change from baseline and measure of variance. If data are not available for each trial arm, we will extract the between-group statistics at the end of treatment. We will consider a minimally important difference of 10 mm (100-mm VAS) between groups [45]. We will extract data from graphs only if the extraction from tables, text, or after contacting authors is not possible. We will manage data in Microsoft Excel and conduct the analyses in R (version 4.0.3) [46].

### Missing Data

We will contact a trial’s corresponding author up to three times via email to request missing data, which will be considered unobtainable if no reply is received within 6 weeks. If data for outcomes of pain and function are not presented in an appropriate form for meta-analysis (such as median and range instead of SDs, standard errors, *t* statistics, or *P* values), we will attempt to impute these using established methods [47,48]. We will conduct sensitivity analyses for pain at end of treatment if we impute missing data for either of these outcomes.

### Assessing Risk of Bias

Two reviewers (RR and one or more reviewers) will independently appraise the risk of bias for each trial using the Cochrane risk of bias tool described in Cochrane Handbook 5.1.0 [41,49]. We will resolve disagreements through discussion or arbitration from a third author (JHM). The Cochrane risk of bias tool assesses the following domains: selection, performance, attrition, detection, reporting, and other sources of bias (eg, conflict of interest, baseline imbalance between groups) [41]. Each domain will receive one of the following judgments: low risk, high risk, or unclear risk of bias. Reviewers will judge items at the study level, which prioritizes information regarding the primary outcome (pain intensity and safety).

### Data Synthesis

We will conduct a quantitative synthesis (meta-analysis) for studies that report sufficient data and compare the medicine targeting neurotrophins versus placebo. We will convert different instruments that measure pain and the associated estimate of precision into a single, most familiar instrument [45]. The meta-analysis will use random-effects models in R [46]. Results will be presented in forest plots. Data from studies that we are not able to use in the quantitative analysis or data from other comparisons (eg, anti-NGF versus tramadol) will be reported narratively.

### Subgroup Analysis

Subgroup analysis will be conducted by condition (sciatica or chronic LBP), duration of pain (acute or chronic), type of medication (eg, anti-NGF versus placebo or only a type of anti-NGF such as tanezumab versus placebo), time point (4 weeks, 12 weeks, 24 weeks, and 48 weeks after the first dose), risk of bias (removing studies classified as having a high risk of bias), and number of participants (excluding studies with less than 10 participants per group).

### Assessment of Heterogeneity

We will evaluate the presence of heterogeneity in the meta-analysis using the Q statistic (α<10%). The magnitude of heterogeneity will be assessed using the estimate of between-study variance (*τ*^2^). We will calculate 95% prediction intervals for pooled effects and interpret prediction intervals spanning greater than 15 points on a 0 to 100 scale on either side of the pooled effect as indicative of important heterogeneity [50]. The distributions of the effect sizes in forest plots will be inspected visually and the I^2^ value will be calculated to indicate the proportion of observed variance due to heterogeneity. Important heterogeneity will be investigated using meta-regression, subgroup analysis, or sensitivity analysis (depending on the availability of data).

### Sensitivity Analysis

We will assess the influence on the effect estimates of the following factors: studies where the definition of the condition is not clear, studies where measures of variance have been imputed, and studies where treatment effects are presented as medians [41,49].

### Extended Funnel Plot

We will construct an extended funnel plot to explore the potential impact of a new trial on the meta-analysis and evaluate whether performing a new trial is worthwhile [51,52].

### Confidence in Cumulative Evidence

Two reviewers (RR and one or more reviewers) will assess the quality of evidence and strength of recommendations. We will use the Grading of Recommendations Assessment Development and Evaluation (GRADE) [53] working group methodology to grade the recommendations. Once this systematic review is limited to include RCTs only, the quality of evidence will be classified as “high” (further research is very unlikely to change our confidence in the estimate of effect) and possibly downgraded to “moderate” (further research is likely to have an important impact on our confidence in the estimate of effect and may change the estimate), “low” (further research is very likely to have an important impact on our confidence in the estimate of effect and is likely to change the estimate), or “very low” (any estimate of effect is very uncertain) [53]. The recommended domains will be assessed using arbitrary percentages that have been used in previous systematic reviews [50].

For risk of bias, we will downgrade the quality of evidence recommendations by one level if >25% but <50% of the participants in our analysis were included in trials that we evaluated to be at “high” risk of bias, and we will downgrade the recommendations by two levels if >50% of the participants were from trials we evaluated to be at “high” risk of bias. For inconsistency, we will downgrade the quality of evidence recommendations by one level if we identify significant heterogeneity. We will assess heterogeneity using the between-study variance parameter (*τ*^2^) and the proportion of study variance not due to sampling error (I^2^). For indirectness, the downgrade will be related to the characteristics of participants in the study. Studies may include participants with sciatica and nonspecific LBP. We will only downgrade the evidence quality if the differences are considered sufficient to make a difference in outcome. For imprecision, we will downgrade the quality of evidence recommendations by one level if the total number of participants is <400 or based on the width of the confidence intervals (for continuous variables as pain intensity and function) by crossing either the null or the threshold for a clinically meaningful effect (10 points on a 0 to 100 scale), and by two levels if the interval spans both. For dichotomous variables (eg, safety), we will downgrade the recommendations by one level if the interval spans the null. For publication bias, we will downgrade the quality of evidence recommendations by a single level if we strongly detect publication bias. We will assess publication bias by visually assessing a funnel plot and by performing a sensitivity analysis. The strength of recommendations will be graded as strong or weak.

## Results

The protocol was registered in Open Science Framework (osf.io/b8adn) on May 19, 2020. As of December 2020, we have identified 1932 records.

## Discussion

There has been a growing number of RCTs investigating the effect of medicines designed to inhibit the nociceptive effect of NGF for osteoarthritis and LBP. This systematic review with meta-analysis will assess the evidence for the efficacy and safety of NGF inhibitors for pain in patients with nonspecific LBP and sciatica. The inclusion of new studies and unpublished data may improve the precision of the effect estimates and guide regulatory actions of the medication for LBP and sciatica.

## Appendix

Multimedia Appendix 1 Search terms.

## Abbreviations

BDNF: brain-derived neurotrophic factor
FDA: US Food and Drug Administration
GDNF: glial cell-derived neurotrophic factor
GRADE: Grading of Recommendations Assessment Development and Evaluation
LBP: low back pain
NGF: nerve growth factor
NRS: numeric rating scale
NSAID: nonsteroidal anti-inflammatory drug
ODI: Oswestry Disability Index
PRISMA-P: Preferred Reporting Items for Systematic Reviews and Meta-Analysis Protocols
RCT: randomized controlled trial
RMDQ: Roland Morris Disability Questionnaire
VAS: visual analog scale
