# Supplementary material for: Efficacy and Safety of Medicines Targeting Neurotrophic Factors in the Management of Low Back Pain: Protocol for a Systematic Review and Meta-analysis
Source: JMIR Res Protoc. 2021 Jan 22;10(1):e22905. doi: 10.2196/22905 (PMC7864772; doi:10.2196/22905)
Supplement: Multimedia Appendix 1 [file resprot_v10i1e22905_app1.docx]

**APPENDIX**

**Search Strategy for MEDLINE (OVID) 1946 to April 03, 2020**

**Part A: Generic search for randomized controlled trials**

1. randomized controlled trial.pt.

2. controlled clinical trial.pt.

3. comparative study.pt.

4. clinical trial.pt.

5. random*.ab.

6. placebo.ab,ti.

7. drug therapy.fs.

8. trial.ab,ti.

9. groups.ab,ti.

10. or/1-9

11. (animals not (humans and animals)).sh.

12. (adolescent* or teen* or youth? or puberty or childhood or children* or p?ediatri* or preschool or pre-school or nursery or kindergarten or infant? or newborn? or neonat* or prematurity or fetal or foetal).mp.

13. 11 or 12

14. 10 not 13

**Part B: Specific search for low back, sacrum and coccyx problems**

15. dorsalgia.ti,ab.

16. exp Back Pain/

17. backache.ti,ab.

18. ((lumb* or back) adj pain).ti,ab.

19. coccydynia.ti,ab.

20. sciatica.ti,ab.

21. spondylosis.ti,ab.

22. lumbago.ti,ab.

23. back disorder$.ti,ab

24. or/15-23

**Part C: Specific search for other spinal disorders**

25. Coccyx.sh

26. Lumbar Vertebrae.sh

27. Intervertebral disc.sh

28. discitis.ti,ab.

29. Sacrum.sh

30. Intervertebral disc degeneration.sh

31. (disc adj degenerat*).ti,ab.

32. (disc adj prolapse*).ti,ab.

33. (disc adj herniat*).ti,ab.

34. spinal fusion.sh.

35. (facet adj joint*).ti,ab.

36. Intervertebral Disc Displacement.sh.

37. postlaminectomy.ti,ab.

38. or/25-37

**Part D: Specific search for interventions of interest**

39. Antibodies, Monoclonal, Humanized/

40. exp Nerve Growth Factors/

41. Tanezumab.mp.

42. fulranumab.mp.

43. fasinumab.mp.

44. anti-NGF.mp.

45. NGF.mp.

46. monoclonal antibodies.mp.

47. ABT-110.mp.

48. PG110.mp.

49. Antinerve growth factor.mp.

50. RN624.mp.

51. REGN475.mp.

52. MEDI-578.mp.

53. exp Interleukins/

54. biological products/

55. ASP-7962.mp.

56. Brain-derived growth factor/

57. neurotrophic.mp.

58. Receptor, trkA/

59. Receptor, trkB/

60. exp Receptors, Nerve Growth Factor/

61. or/39-60

**Part E. Combination of terms**

62. 24 or 38 (all back pain)

63. 62 and 61 (all back pain and all interventions of interest)

69. 14 and 63 (all RCTs of interventions of interest in back pain)
